# Supplementary material for: Yu-Shiba-Rusinov bands in a self-assembled kagome lattice of magnetic molecules
Source: Nat Commun. 2024 Aug 1;15:6474. doi: 10.1038/s41467-024-50829-5 (PMC11291492; doi:10.1038/s41467-024-50829-5)
Supplement: Supplementary file 1 — Supplementary Information [file 41467_2024_50829_MOESM1_ESM.pdf]

Supplementary Information

Laëtitia Farinacci,[1] Gaël Reecht,[1] Felix von Oppen,[2] and Katharina J. Franke[1,1]
[1]*Fachbereich Physik, Freie Universität Berlin, Arnimallee 14, 14195 Berlin, Germany.*[*]
[2]*Dahlem Center for Complex Quantum Systems and Fachbereich Physik, Freie Universität Berlin, 14195 Berlin, Germany*

Supplementary Note 1. IDENTIFICATION OF DE-CHLORINATED FEP MOLECULES

In the main text, we described the self-assembly of de-chlorinated FeP molecules and Cl atoms. We argued that the Cl atoms detached from the FeP-Cl molecules upon adsorption, and that the amount of Cl atoms on the surface, and thus the amount, which is available to participating in the self-assembly, can be tuned by the annealing temperature. Yet, we found that the dechlorinated molecules can appear with two slightly different apparent heights in the STM images. The brighter molecules (clover shape) may - at first sight - be interpreted as the chlorinated species. While our systematic analysis with increasing temperature as described in the main text already suggests that both types of molecules are de-chlorinated, we provide another point of evidence that the types "only" exhibit different electronic properties here.

In Supplementary Figure 1a we show an STM image recorded at low bias voltage, where two types of molecules appear with different height (clover shape with a central protrusion and square-like). An image taken at larger bias voltage of the same area reveals all molecules with identical appearance (Supplementary Figure 1b), suggesting that

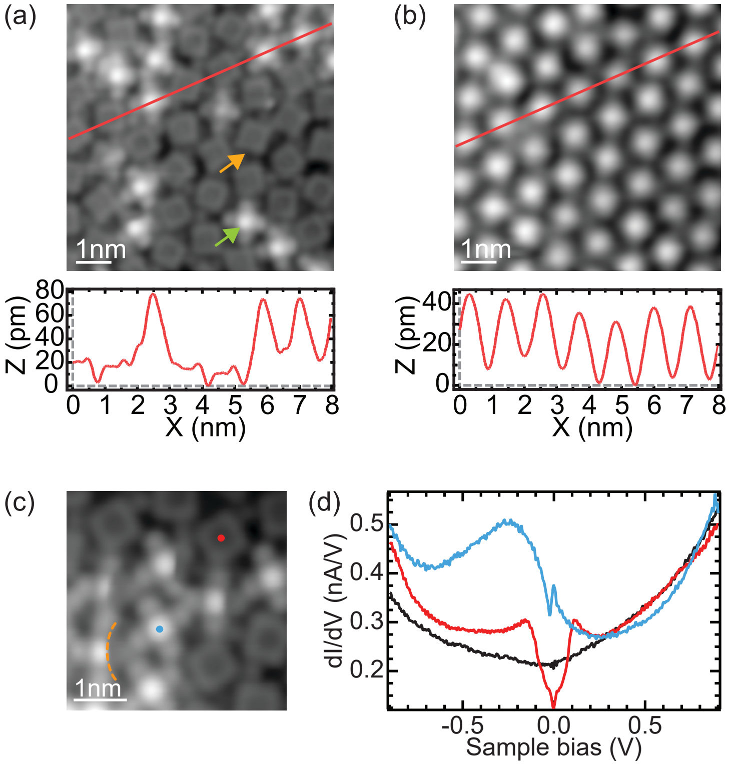

Supplementary Figure 1. (a) Topography image recorded with $V_{\rm bias} = 5$ mV, $I = 200$ pA in which two types of molecules can be identified (see green and orange arrows), as exemplified by the height profile taken along the red line. (b) Topography image of the same area recorded with $V_{\rm bias} = 700$ mV, $I = 200$ pA: the molecules now have the same appearance as illustrated by the corresponding line profile. (c) Topography images ($V_{\rm bias} = 5$ mV, $I = 200$ pA) where the two types of molecules can be identified – a few molecules display both appearances (see orange dashed line). The dots indicate the locations of the dI/dV spectra in (d). (d) dI/dV spectra recorded above the centers of a clover-shaped molecule (blue) and a square-shaped molecule (red) along with a reference on bare Pb (black). Feedback opened at $V_{\rm bias} = 900$ mV, $I = 300$ pA and signal modulated with $V_{\rm rms} = 5$ mV.

none of the molecules carries a Cl ligand attached to the Fe center. The different electronic structure leading to different appearance in the STM image is also reflected in their different dI/dV spectra. Molecules, which appear clover-shape and bright at low bias, exhibit a broad resonance around -300 mV, whereas the dark square-like molecules exhibit a broad pair of symmetric steps around the Fermi energy. Note also that in Supplementary Figure 1c some molecules have a hybrid appearance: clover-shaped on the left and central part, square-shaped on the right (the orange dashed line serves as a guide to the eye). We speculate that the presence of Cl adatoms effectively gates the electronic properties of the FeP molecules in their vicinity, which leads to these two different molecular types.

In very rare cases, we also observe molecules with a very bright protrusion above their center ($< 1\%$), as shown in the supplementary information of [1]. They exhibit two pairs of resonances outside the superconducting gap in dI/dV spectra, which are a fingerprint of spin excitations for magnetic adsorbates on superconductors. As a matter of fact, their dI/dV spectra is almost identical to that of iron-octa-ethyl-porphyrin-chloride (FeOEP-Cl) molecules on Pb(111) [2]. FeOEP and FeP molecules only differ by the presence of additional ligands for FeOEP, away from the Fe center. Since the oxidation state, spin state and magnetic anisotropy of Fe is mostly dictated by its direct surroundings, we conclude that the molecules with a very high protrusion have kept their Cl ligand upon adsorption. The two types of molecules presented in the main text are thus assigned to FeP molecules, and the small protrusions in between them to Cl adatoms.

Supplementary Note 2. YSR STATES AT THE EDGE OF KAGOME PRECURSORS

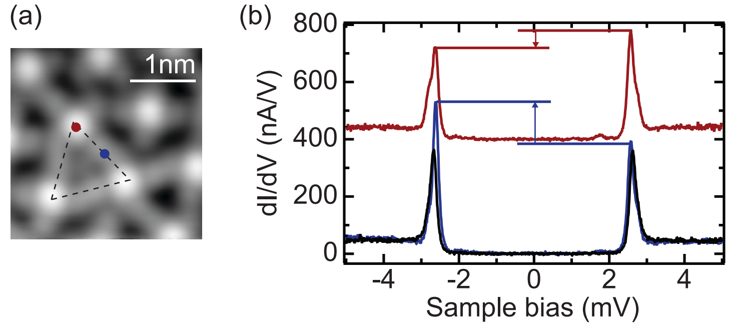

Supplementary Figure 2. (a) Topography image of a single tile made of three FeP molecules and one Cl adatom ($V_{\mathrm{bias}} =$ 5 mV, $I = 200$ pA). (b) dI/dV spectra taken above the center (red – offset for clarity) and ligand (blue) of a FeP molecule as indicated in (a), along with a reference spectrum on bare Pb (black). Feedback opened at $V_{\mathrm{bias}} = 5$ mV, $I = 200$ pA and signal modulated with $V_{\mathrm{rms}} = 15$ μeV.

In the main paper we show evidence of hybridization between YSR states induced by molecules surrounded by two Cl adatoms. The presence of these two adatoms is crucial for the YSR hybridization. Indeed, when there is only one Cl adatom in the vicinity, the FeP molecules do not display any YSR state well inside the gap but rather an asymmetry in the intensity of the coherence peaks (Fig. 3b of the main text and Supplementary Figure 2). The opposite intensity asymmetry above the Fe center and molecular ligand is consistent with the presence of a YSR state close to the gap edge with the asymmetry being a result of different interfering tunneling paths [3]. The absence of splitting in the dI/dV spectra indicates that, if any hybridization takes place between molecules surrounded by only one Cl adatom, it remains below our energy resolution and at a different energy that those that pertain to the YSR hybridization described in the main paper.

Supplementary Note 3. DECONVOLUTION OF dI/dV SPECTRA AND FITTING OF THE SAMPLE LDOS

In order to investigate the distribution of the hybridized YSR states it is important to remove the influnce of the density of states (DOS) of the tip. Indeed, the non-flat DOS of the superconducting tip can lead to negative differential resistance effects that would artificially lower the intensity of a YSR state in close vicinity of another one.

We perform a numerical deconvolution of the dI/dV spectra following the approach of Pillet et al.[4]. Neglecting proportionality constants, the tunneling current is given by:

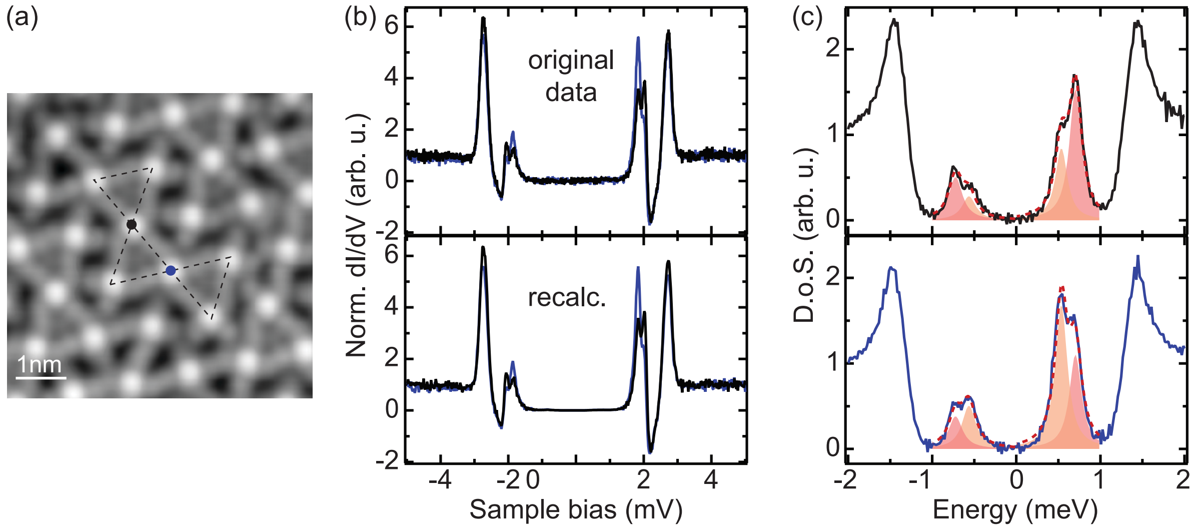

Supplementary Figure 3. (a) Topography image of a kagome precursor ($V_{\rm bias} = 5$ mV, $I = 200$ pA). (b) Upper graph: dI/dV spectra taken at the locations indicated in (a) (feedback opened at $V_{\rm bias} = 5$ mV, $I = 200$ pA and signal modulated with $V_{\rm rms} = 15$ μeV). Lower graph: reconvolved spectra. The match between the two graphs ensures the consistency of the deconvolution procedure. (c) Sample DOS obtained by deconvolving the dI/dV spectra in (b). Red dashed lines show fits with a sum of Lorentzian peaks for both bias polarities, the contribution of each Lorentzian being indicated by filled curves.

$$I = \int [f(\omega - eV) - f(\omega)]\rho_t(\omega - eV)\rho_s(\omega)d\omega, \tag{1}$$

where f is the Fermi function, ρ_s (ρ_t) the DOS of the sample (tip) and V the bias voltage. As a result, the differential conductance is the sum of two integrals.

$$\frac{dI}{dV} = \int -e[f(\omega - eV) - f(\omega)] \left.\frac{\partial \rho_t}{\partial E}\right|_{E=\omega-eV} \rho_s(\omega)d\omega + \int -e \left.\frac{\partial f}{\partial E}\right|_{E=\omega-eV} \rho_t(\omega - eV)\rho_s(\omega)d\omega. \tag{2}$$

These two integrals are of the form $\int K(\omega)\rho_s(\omega)d\omega$ where

$$K_1(\omega) = -e[f(\omega - eV) - f(\omega)] \left.\frac{\partial \rho_t}{\partial E}\right|_{E=\omega-eV} \tag{3}$$

$$K_2(\omega) = -e \left.\frac{\partial f}{\partial E}\right|_{E=\omega-eV} \rho_t(\omega - eV). \tag{4}$$

They can be numerically approximated via a discretization of the energy and bias voltage so that the differential conductance is simply given by a matrix product:

$$\frac{dI}{dV} = K \cdot \rho_s, \tag{5}$$

where $K = K_1 + K_2$ depends only on the tip DOS that we assume to be Bardeen-Cooper-Schrieffer (BCS) like. The sample DOS is then obtained by pseudo-inverting the matrix K:

$$\rho_s = K^{-1} \cdot \frac{dI}{dV}. \tag{6}$$

To ensure the consistency of the deconvolution procedure, we reconvolve the resulting DOS with the K matrix and verify that the obtained spectrum corresponds to the original $\mathrm{d}I/\mathrm{d}V$ spectrum. Two examples of this are shown in Supplementary Figure 3. Subsequently, we fit the sample DOS with a sum of Lorentzians. The amplitude of each Lorentzian indicates the amplitude of the corresponding YSR state at the position where the spectrum was taken. In the main text, we only display the amplitude of the fits at positive energies, but we ensured that the negative-energy results show the same patterns.

Supplementary Note 4. IDENTIFICATION OF THE YSR GROUND STATE

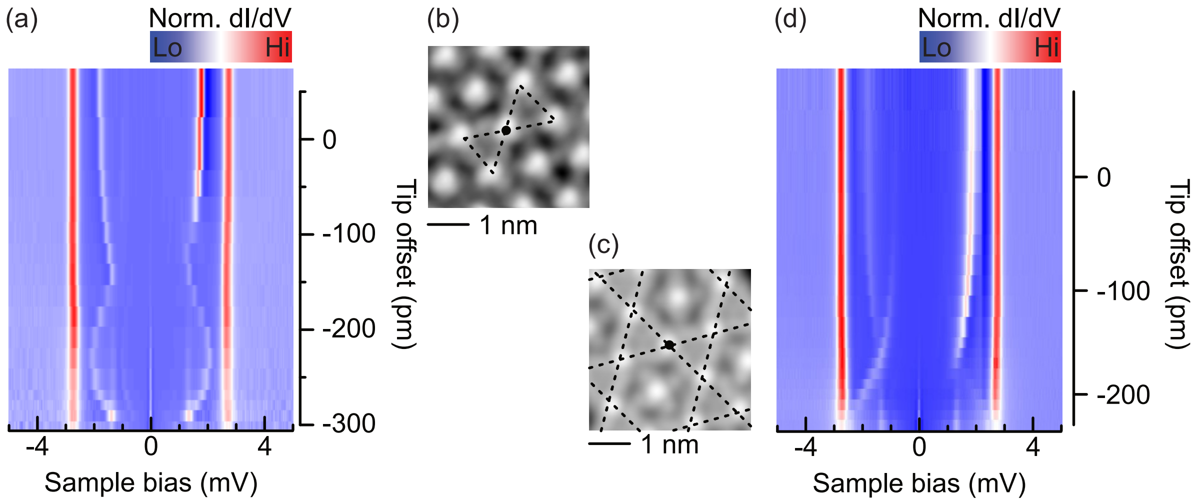

Supplementary Figure 4. (a) $\mathrm{d}I/\mathrm{d}V$ spectra measured above the center of a Kagome precursor as shown in (b) ($V_{\mathrm{bias}} = 5$ mV, $I = 200$ pA) as a function of tip offset after opening the feedback with $V_{\mathrm{bias}} = 5$ mV, $I = 200$ pA (negative offsets defined as bringing the tip closer to the surface, signal modulated with $V_{\mathrm{rms}} = 15\mu$ eV). (c) Topography image ($V_{\mathrm{bias}} = 5$ mV, $I = 200$ pA) in which a kagome lattice is formed, as indicated by black dashed lines. A black circle indicates the location where the data set of (d) has been recorded. (d) $\mathrm{d}I/\mathrm{d}V$ spectra as a function of tip offset after opening the feedback at $V_{\mathrm{bias}} = 5$ mV, $I = 200$ pA (signal modulated with $V_{\mathrm{rms}} = 20\mu$eV).

In order to identify the ground state of the system, one can perform a tip approach toward the molecule to modulate the exchange coupling strength of the magnetic impurity to the substrate [1, 5]. The molecule studied here is the same as in [1], yet in a different molecular environment. Starting with a set point of 5mV, 200pA the approach first takes place in the attractive regime. We perform such tip approach above a molecule that is surrounded by two Cl adatoms but without YSR hybridization (Supplementary Figure 4a-b) as well as above a molecule that is inside a Kagome domain (Supplementary Figure 4c-d). In both cases, the results are in line with those previously obtained above a molecule at the edge of a kagome lattice [1]. As the tip is brought closer to the Fe center, the YSR states shifts towards Fermi energy and we observe a crossing of the quantum phase transition for tip offsets between -100 pm and -200 pm, from there on the YSR state shifts away from the Fermi energy. This shift of the YSR state is attributed to a weakening of the exchange coupling between the Fe center and substrate as the molecule is pulled toward the tip due to attractive van-der-Waals interactions. Going even closer in Supplementary Figure 4a, we observe a reversed trend starting from $\Delta z \sim 240$ pm, with the YSR shifting toward the Fermi energy. This corresponds to the onset of the repulsive regime where the molecule is pushed back toward the Pb substrate.

All in all, these results, with in particular the shift toward the Fermi energy at large tip-sample distance, indicate that the YSR state of the molecules is in the screened regime. Note that for the experiment shown in Supplementary Figure 4c-d, the YSR state is gradually detuned from its neighbours with diminishing hybridization upon tip approach.

Supplementary Note 5. KONDO EFFECT IN THE NORMAL STATE OF PB

In the normal state of Pb, the FeP molecules display a Kondo resonance. In Supplementary Figure 5b, we show the $\mathrm{d}I/\mathrm{d}V$ spectra taken above the center and molecular ligand of the four molecules of Fig.6 of the main text (see

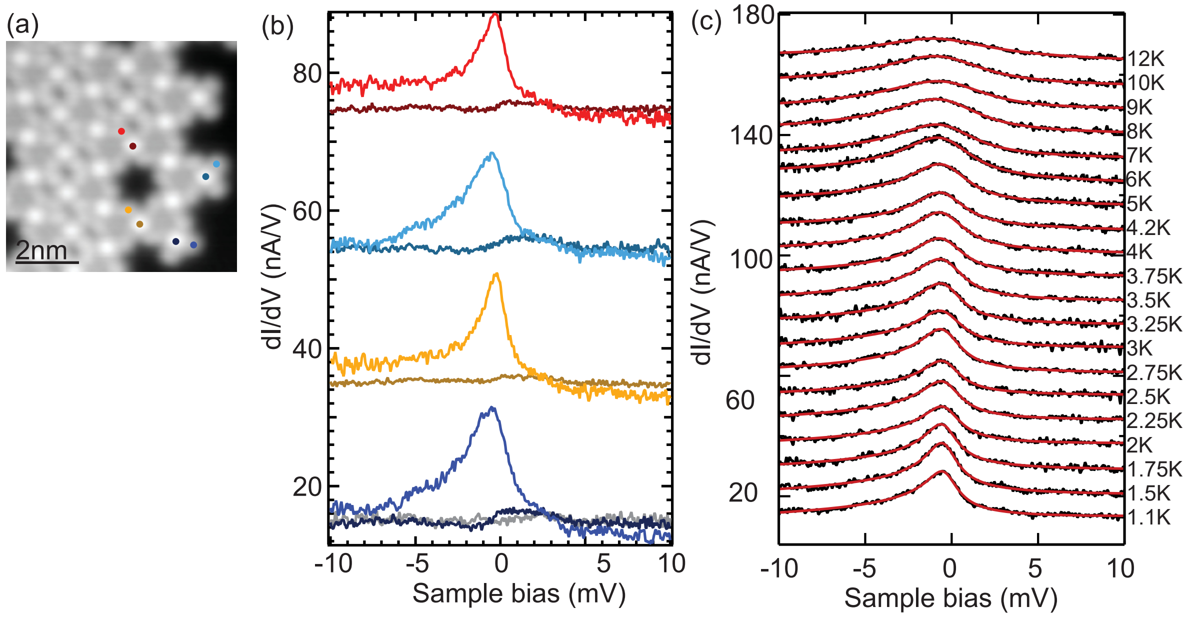

Supplementary Figure 5. (a) Topography image of the molecules investigated in Fig.6 of the main text ($V_{\mathrm{bias}} = -45$ mV, $I = 30$ pA). (b) $\mathrm{d}I/\mathrm{d}V$ spectra taken at the location indicated in (a) (feedback opened at $V_{\mathrm{bias}} = 10$ mV, $I = 150$ pA and signal modulated with $V_{\mathrm{rms}} = 50$ μeV). A reference spectrum taken above bare Pb is shown in grey, spectra are offset for clarity. (c) Evolution with temperature of the Kondo resonance above the ligand of the lowest molecule in a (dark blue) with Fano-Hurwitz fits in red [6].

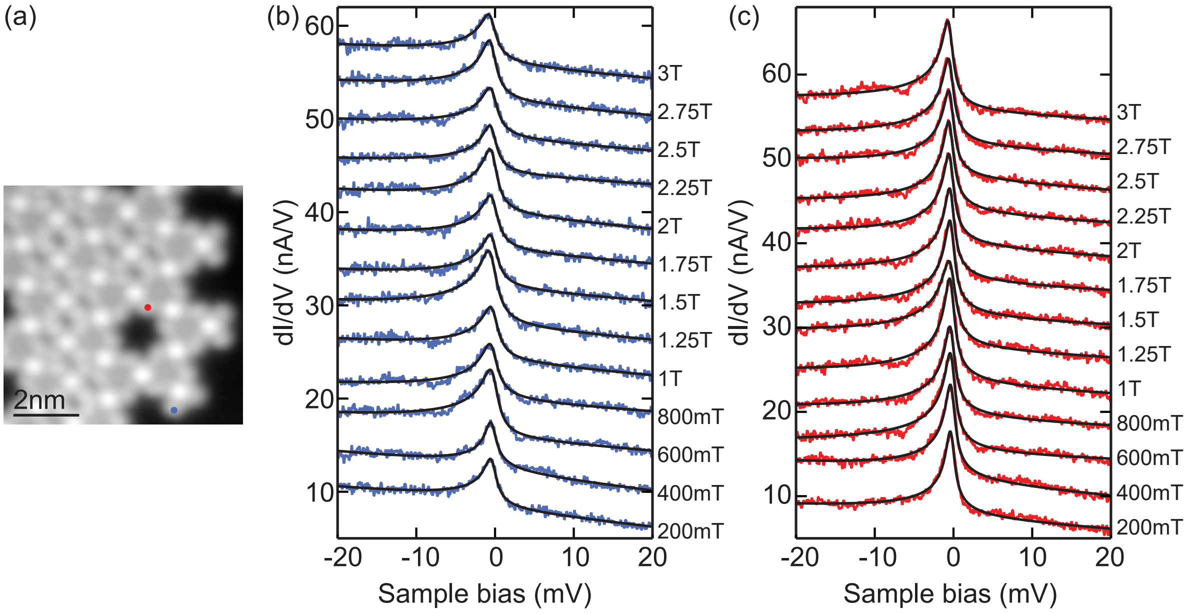

Supplementary Figure 6. (a) Topography image ($V_{\mathrm{bias}} = -45$ mV, $I = 30$ pA) of the island shown in Fig.6 of the main text with the two locations where the magnetic field dependent measurements where taken. The two data sets along with the Fano-Hurwitz fits [6] (black curves) are displayed in (b) and (c) (feedback opened at $V_{\mathrm{bias}} = 10$ mV, $I = 150$ pA and signal modulated with $V_{\mathrm{rms}} = 50$ μeV).

Supplementary Figure 5a).

Due to interferences between tunneling paths [3], the lineshape of the Kondo resonance is very different between the center and ligand of the molecules. The resonances are more intense and better resolved above the molecular

ligands. We therefore use these positions to study the evolution of the resonance with temperature (Supplementary Figure 5) and external magnetic field (Supplementary Figure 6). In Supplementary Figure 5c we show an exemplary set of data taken at various temperatures above the molecule at the edge of the island in Supplementary Figure 5a, indicated by a dark blue circle. Each spectrum is fitted with a Fano-Hurwitz function [6] to extract the half-width at half-maximum (HWHM) of the Kondo resonance. Similar data sets were taken at the ligand positions of other molecules marked in the topography image of Supplementary Figure 5a. The fit results are presented in Fig.6b of the main text, where the error bars correspond to the standard deviation of the fits.

In Supplementary Figure 6 we show the evolution of the Kondo resonance with external magnetic field for two molecules, one at the edge and one inside the island, as indicated in the topography image of Supplementary Figure 6a. For both molecules, the linewidth – extracted by Fano-Hurwitz fits shown in black – stays constant over the range of external magnetic fields achievable in our experiment.

Overall, these results indicate a smaller coupling to the underlying substrate of the molecules inside the island than those at the edge. This is in line with the fact that these molecules exhibit YSR states at different energies in the superconducting state of the sample. Kondo width and magnetic field dependence show that the molecules lie in the strong-coupling Kondo regime with a critical field above 3 T.

The temperature and B-field behavior are in agreement with a single spin coupled to the substrate. This together with the absence of any deviation from a Fano-Hurwitz lineshape suggests that any Kondo-lattice behavior is weak and beyond our resolution.

Supplementary Note 6. KAGOME LATTICE OF YU-SHIBA-RUSINOV STATES

In the normal state, the tight-binding band structure of a kagome lattice contains a flat band, when restricting to nearest-neighbor hopping. A kagome lattice formed by YSR states is likely to change this in two ways. First, YSR states are only power-law localized except at very large lengths of the order of the superconducting coherence length [7]. This makes it plausible that next-nearest-neighbor hopping needs to be retained. (We have checked that next-nearest-neighbor coupling of the strength assumed in Supplementary Figure 7 below is entirely consistent with our experimental results for the YSR trimer.) Second, due to the doubling of states in the Bogoliubov-de Gennes description, all normal-state bands appear twice in a symmetric manner about the center of the superconducting gap. Pairing in principle couples these bands, which may also modify the simple kagome band structure. Here, we show that next-nearest-neighbor hopping and pairing effects broaden the flat band of the kagome band structure. We assume that the adatom spins are polarized, choosing a ferromagnetic arrangement for simplicity. Without spin-orbit coupling and assuming s-wave pairing of the substrate superconductor, hybridization of the subgap YSR states yields uncoupled sets of electron and hole bands. Each of these contains a flat band provided that the hybridization of YSR states is isotropic and restricted to nearest neighbors [8]. Pairing effects come into play, when accounting for spin-orbit coupling, which introduces p-wave pairing [7, 9]. Assuming Rashba coupling, this pairing is of chiral p-wave nature. Restricting to nearest-neighbor and next-nearest-neighbor hybridization of the YSR states, the Bogoliubov-de Gennes Hamiltonian takes the form

$$H(\mathbf{k}) = \begin{pmatrix} H_n(\mathbf{k}) & \Delta(\mathbf{k}) \\ \Delta^\dagger(\mathbf{k}) & -[H_n(-\mathbf{k})]^T \end{pmatrix}. \tag{7}$$

Here, the normal-state Hamiltonian is $H_n = H_{nn} + H_{nnn}$ with

$$H_{nn}(\mathbf{k}) = \begin{pmatrix} \epsilon_{\rm YSR} & -t(1+e^{-i\mathbf{k}\cdot\mathbf{a}_1}) & -t(1+e^{i\mathbf{k}\cdot\mathbf{a}_2}) \\ -t(1+e^{i\mathbf{k}\cdot\mathbf{a}_1}) & \epsilon_{\rm YSR} & -t(1+e^{-i\mathbf{k}\cdot\mathbf{a}_3}) \\ -t(1+e^{-i\mathbf{k}\cdot\mathbf{a}_2}) & -t(1+e^{i\mathbf{k}\cdot\mathbf{a}_3}) & \epsilon_{\rm YSR} \end{pmatrix} \tag{8}$$

accounting for YSR energy $\epsilon_{\rm YSR}$ and nearest-neighbor hopping and

$$H_{nnn}(\mathbf{k}) = \begin{pmatrix} 0 & -t'(e^{i\mathbf{k}\cdot\mathbf{a}_2}+e^{i\mathbf{k}\cdot\mathbf{a}_3}) & -t'(e^{-i\mathbf{k}\cdot\mathbf{a}_3}+e^{-i\mathbf{k}\cdot\mathbf{a}_1}) \\ -t'(e^{-i\mathbf{k}\cdot\mathbf{a}_2}+e^{-i\mathbf{k}\cdot\mathbf{a}_3}) & 0 & -t'(e^{i\mathbf{k}\cdot\mathbf{a}_1}+e^{i\mathbf{k}\cdot\mathbf{a}_2}) \\ -t'(e^{i\mathbf{k}\cdot\mathbf{a}_3}+e^{i\mathbf{k}\cdot\mathbf{a}_1}) & -t'(e^{-i\mathbf{k}\cdot\mathbf{a}_1}+e^{-i\mathbf{k}\cdot\mathbf{a}_2}) & 0 \end{pmatrix} \tag{9}$$

accounting for next-nearest neighbor hopping. t and t' denote the amplitudes of nearest-neighbor and next-nearest-neighbor hopping, respectively. Both hoppings are assumed isotropic. The vectors $\mathbf{a}_1 = 2a[1,0]$, $\mathbf{a}_2 = 2a[\cos(2\pi/3), -\sin(2\pi/3)]$, and $\mathbf{a}_3 = 2a[\cos(2\pi/3), \sin(2\pi/3)]$ are given in terms of the bond length a. The pairing

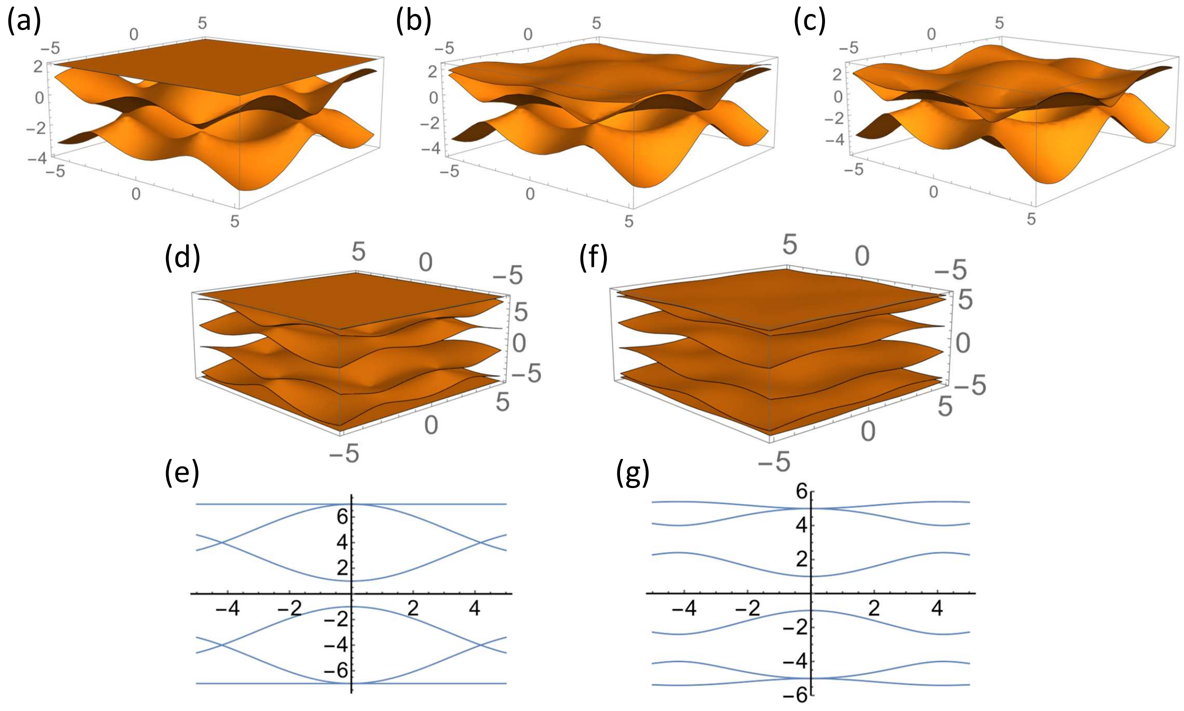

Supplementary Figure 7. Broadening of the Kagome flat band by next-nearest-neighbor hopping and pairing interactions. (a) Normal-metal band structure as a function of k_x and k_y for a tight-binding model on a kagome lattice with nearest neighbor hopping only ($t' = 0$), exhibiting a flat band. (b) Same with next-nearest-neighbor hopping $t' = 0.2$, which leads to a finite dispersion of the flat band. The dispersion carries over to the spectrum of the Bogoliubov-de Gennes equation. (c) Same as (b) with $t' = 0.4$. (d) Bogoliubov-de Gennes spectrum for a Kagome lattice with nearest neighbor hopping and without pairing, showing the doubling of the bands. Here, the YSR energy is chosen to be $\epsilon_{\rm YSR} = 5$. (e) Linecut through band structure as a function of k_x for $k_y = 0$, highlighting the flat electron and hole bands. (f) Bogoliubov-de Gennes spectrum for a Kagome lattice with nearest neighbor hopping and pairing strength $\Delta_p = 1$ for $\epsilon_{\rm YSR} = 3$. (g) Corresponding linecut as a function of k_x for $k_y = 0$, highlighting the broadening of the flat bands by pairing.

matrix takes the form

$$\Delta(\mathbf{k}) = \begin{pmatrix} 0 & -\Delta_p e^{-i2\pi/3}(1 - e^{-i\mathbf{k}\cdot\mathbf{a}_1}) & \Delta_p e^{i2\pi/3}(1 - e^{i\mathbf{k}\cdot\mathbf{a}_2}) \\ \Delta_p e^{i2\pi/3}(1 - e^{i\mathbf{k}\cdot\mathbf{a}_1}) & 0 & -\Delta_p(1 - e^{-i\mathbf{k}\cdot\mathbf{a}_3}) \\ -\Delta_p e^{-i2\pi/3}(1 - e^{-i\mathbf{k}\cdot\mathbf{a}_2}) & \Delta_p(1 - e^{i\mathbf{k}\cdot\mathbf{a}_3}) & 0 \end{pmatrix} \tag{10}$$

with induced pairing strength Δ_p. (Here, we neglect next-nearest-neighbor pairing, since even nearest-neighbor pairing effects are a correction to the band structure.)

We diagonalize $H(\mathbf{k})$ and show results in Supplementary Figure 7. We observe that the flat band broadens significantly in the presence of next-nearest-neighbor hopping [Supplementary Figure 7(b,c)] or pairing [Supplementary Figure 7(f,g)]. The magnitude of the pairing effect depends on the ratio of Δ_p/t. (Here, Δ_p should not be confused with the gap of the host superconductor, which merely provides an upper limit for the strength of Δ_p.) The value of Δ_p is controlled by the strength of spin-orbit coupling. Thus, when restricting to nearest-neighbor hopping, the YSR band structure will contain essentially flat electron and hole bands in the limit of weak spin-orbit coupling, while the flat-band nature is progressively lost with increasing spin-orbit coupling. In experiment, both next-nearest-neighbor hopping and pairing are expected to contribute to the band structure. The similarities in their phenomenology makes it difficult to disentangle the two effects based on the available data. We expect these conclusions to remain valid beyond the simplifying assumptions (ferromagnetic order, nearest-neighbor hybridization, single pair of YSR resonances) made here.

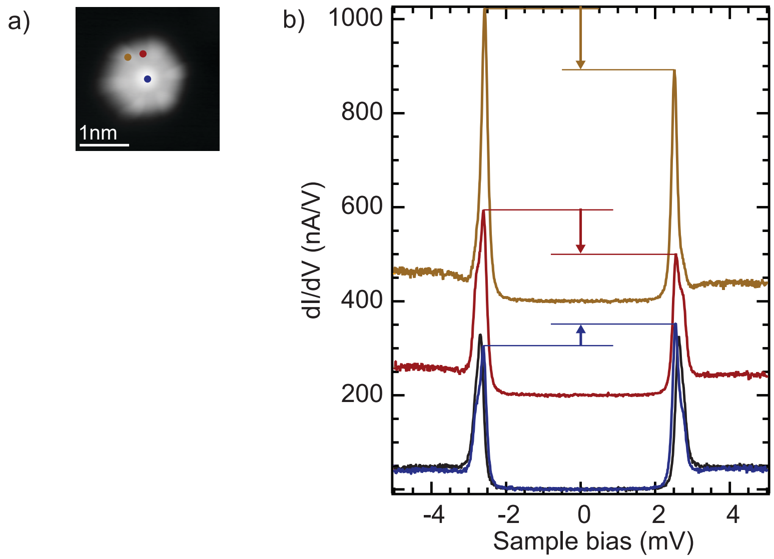

Supplementary Figure 8. (a) Topography images of a molecule dragged out of an island ($V_{\text{bias}} = 5$ mV, $I = 200$ pA). (b) dI/dV spectra recorded with a superconducting tip above the positions shown in a and above the bare Pb surface (black) (feedback opened at $V_{\text{bias}} = 5$ mV, $I = 200$ pA and signal modulated with $V_{\text{rms}} = 15$ μeV, spectra are offset for clarity.

Supplementary Note 7. ISOLATED FEP MOLECULE ON PB(111)

After dragging a FeP molecule out of an island we find that it rotates between three equivalent adsorption sites on the Pb(111) surface as seen by the three-fold symmetry of the topography image in Supplementary Figure 8. The dI/dV spectra recorded above the molecule show an asymmetry of the coherence peaks which we attribute to the presence of a YSR state close to the gap edge. This asymmetry is in agreement with the molecules found in the pores of the kagome lattice - which have no Cl in their vicinity - as shown in the main paper (blue spectrum Fig.5d of the main text).

* laetitia.farinacci@polytechnique.org

[1] L. Farinacci, G. Ahmadi, G. Reecht, M. Ruby, N. Bogdanoff, O. Peters, B. W. Heinrich, F. von Oppen, and K. J. Franke, Tuning the coupling of an individual magnetic impurity to a superconductor: Quantum phase transition and transport, Phys. Rev. Lett. **121**, 196803 (2018).

[2] B. W. Heinrich, L. Braun, J. I. Pascual, and K. J. Franke, Protection of excited spin states by a superconducting energy gap, Nat. Phys. **9**, 765 (2013).

[3] L. Farinacci, G. Ahmadi, M. Ruby, G. Reecht, B. W. Heinrich, C. Czekelius, F. von Oppen, and K. J. Franke, Interfering tunneling paths through magnetic molecules on superconductors: Asymmetries of Kondo and Yu-Shiba-Rusinov resonances, Phys. Rev. Lett. **125**, 256805 (2020).

[4] J. Pillet, C. Quay, P. Morfin, C. Bena, A. L. Yeyati, and P. Joyez, Andreev bound states in supercurrent-carrying carbon nanotubes revealed, Nat. Phys. **6**, 965 (2010).

[5] L. Malavolti, M. Briganti, M. Hänze, G. Serrano, I. Cimatti, G. McMurtrie, E. Otero, P. Ohresser, F. Totti, M. Mannini, R. Sessoli, and S. Loth, Tunable spin-superconductor coupling of spin 1/2 vanadyl phthalocyanine molecules, Nano Lett. **18**, 7955 (2018).

[6] E. Turco, M. Aapro, S. C. Ganguli, N. Krane, R. Drost, N. Sobrino, A. Bernhardt, M. Juríček, R. Fasel, P. Ruffieux, *et al.*, Accurate Kondo temperature determination of spin-1/2 magnetic impurities, arXiv preprint arXiv:2310.09326 (2023).

[7] F. Pientka, L. I. Glazman, and F. von Oppen, Topological superconducting phase in helical Shiba chains, Phys. Rev. B **88**, 155420 (2013).

[8] The symmetry of the hybridization will in general depend on the YSR wave function of the monomer [**?**]. It is plausible that the YSR states are associated with the d_{z^2} orbital of the magnetic atoms, suggesting isotropic YSR states.

[9] J. Röntynen and T. Ojanen, Topological Superconductivity and High Chern Numbers in 2D Ferromagnetic Shiba Lattices, Phys. Rev. Lett. **114**, 236803 (2015).
